# *In silico* analysis of angiotensin-converting enzyme inhibitory compounds obtained from soybean [*Glycine max* (L.) Merr.]

**DOI:** 10.3389/fphys.2023.1172684

**Published:** 2023-05-31

**Authors:** Ayyagari Ramlal, Isha Bhat, Aparna Nautiyal, Pooja Baweja, Sahil Mehta, Vikash Kumar, Shikha Tripathi, Rohit Kumar Mahto, Manisha Saini, Bingi Pujari Mallikarjuna, Shukla Saluja, S. K. Lal, Sreeramanan Subramaniam, Iten M. Fawzy, Ambika Rajendran

**Affiliations:** ^1^ Division of Genetics, ICAR-Indian Agricultural Research Institute (IARI), Pusa Campus, New Delhi, India; ^2^ School of Biological Sciences, Universiti Sains Malaysia (USM), Georgetown, Penang, Malaysia; ^3^ Department of Biosciences, Jamia Millia Islamia, New Delhi, Delhi, India; ^4^ Department of Botany, Deshbandhu College, University of Delhi, New Delhi, India; ^5^ Department of Botany, Maitreyi College, University of Delhi, New Delhi, India; ^6^ Department of Botany, Hansraj College, University of Delhi, New Delhi, India; ^7^ Faculty of Agricultural Sciences, Institute of Applied Sciences and Humanities, GLA University, Mathura, Uttar Pradesh, India; ^8^ ICAR- National Institute for Biotechnology, New Delhi, India; ^9^ Department of Botany, Institute of Science, Banaras Hindu University (BHU), Varanasi, Uttar Pradesh, India; ^10^ School of Biotechnology, Institute of Science, Banaras Hindu University (BHU), Varanasi, Uttar Pradesh, India; ^11^ Division of Genetics, Indian Council of Agricultural Research (ICAR)-Indian Agricultural Research Institute (IARI), Regional Research Centre, Dharwad, Karnataka, India; ^12^ Department of Botany, Sri Venkateswara College, University of Delhi, New Delhi, India; ^13^ Chemical Centre Biology (CCB), Universiti Sains Malaysia (USM), Bayan Lepas, Penang, Malaysia; ^14^ Institute of Nano Optoelectronics Research and Technology, Universiti Sains Malaysia (USM), Bayan Lepas, Penang, Malaysia; ^15^ Department of Pharmaceutical Chemistry, Faculty of Pharmacy, Future University in Egypt, Cairo, Egypt

**Keywords:** angiotensin-converting enzyme I, beta-sitosterol, cardiovascular diseases, natural drugs, renin–angiotensin–aldosterone system, phytocompounds, phytic acid, soybean

## Abstract

Cardiovascular diseases (CVDs) are one of the major reasons for deaths globally. The renin–angiotensin–aldosterone system (RAAS) regulates body hypertension and fluid balance which causes CVD. Angiotensin-converting enzyme I (ACE I) is the central Zn-metallopeptidase component of the RAAS playing a crucial role in maintaining homeostasis of the cardiovascular system. The available drugs to treat CVD have many side effects, and thus, there is a need to explore phytocompounds and peptides to be utilized as alternative therapies. Soybean is a unique legume cum oilseed crop with an enriched source of proteins. Soybean extracts serve as a primary ingredient in many drug formulations against diabetes, obesity, and spinal cord-related disorders. Soy proteins and their products act against ACE I which may provide a new scope for the identification of potential scaffolds that can help in the design of safer and natural cardiovascular therapies. In this study, the molecular basis for selective inhibition of 34 soy phytomolecules (especially of beta-sitosterol, soyasaponin I, soyasaponin II, soyasaponin II methyl ester, dehydrosoyasaponin I, and phytic acid) was evaluated using *in silico* molecular docking approaches and dynamic simulations. Our results indicate that amongst the compounds, beta-sitosterol exhibited a potential inhibitory action against ACE I.

## 1 Introduction

The angiotensin-converting enzyme (ACE) (EC 3.4.15.1) is a chloride-dependent and zinc-containing peptidyl-dipeptidase A enzyme (Natesh et al., 2003; Sharma et al., 2016). It is a crucial enzyme that regulates the formation of angiotensin I (Ang I) to angiotensin II (Ang II) and, in turn, blood pressure (BP). This enzyme is a part of the renin–angiotensin–aldosterone system (RAAS). ACE inhibitors have a therapeutic role in regulating the level of blood pressure and, thus, preventing cardiovascular diseases (CVDs) (Zhao et al., 2019; Tahir et al., 2020; Kircheva et al., 2021; Li et al., 2022). CVD is one of the major diseases and a leading cause of mass mortality estimating around 17.9 million deaths each year (Roth et al., 2020). Hypertension is one of the primary reasons for CVD affecting the vital organs including the brain and kidneys. Several other pathophysiological processes also occur simultaneously along with hypertension which include the stiffening of large ducts (aorta and carotid artery) and elastic artery, smooth muscle cell proliferation, vasoconstriction, and dysfunction of the endothelium (Agrawal et al., 2016; Margalef et al., 2017). The RAAS helps in the regulation of fluid balance and plays a crucial part in maintaining homeostasis of the cardiovascular system and normalizing BP. The inhibitors of ACE competitively inhibit its conversion to Ang II which is formed by the ACE I from Ang I. Thus, formed Ang II then stimulates the release of aldosterone, which eventually elevates BP (Atlas, 2007; Shimakage et al., 2012; Kircheva et al., 2021) and also simultaneously catalyzes the degradation of bradykinin, a potent vasodilator (Margalef et al., 2017). The process of controlling BP is a complex mechanism involving a cascade of steps and different organs, involving the autonomic nervous system (ANS), vasopressor and vasodepressor hormones, the total volume of body fluid, renal function, and vasculature. The endothelium is directly involved in controlling BP by producing multiple vasodilators and vasoconstrictors such as nitric oxide (NO), which is the most important endothelial vasodilator factor (Margalef et al., 2017; Khan and Kumar, 2019; Chamata et al., 2020).

The commercially available synthetic ACE inhibitors (enalapril, lisinopril, etc.) cause side effects like nausea, hyperkalemia, headache, swelling of the lower portion of the skin, cough, disturbances in taste, and angioneurotic edema (Sharma et al., 2016). Various plants have been reported to have potential ACE inhibition properties as reviewed by Patten et al. (2016). The plant-based bioactive compounds are better alternatives to synthetic drugs because they are non-toxic and easily available and have no side effects (Patten et al., 2016; Khan and Kumar, 2019; Chamata et al., 2020; Zhang et al., 2022). Therefore, there is a need to switch and find alternative natural sources (like medicinal crops) having promising health-promoting benefits with no side effects (Hermida et al., 2011; Sitanggang et al., 2021; Xu et al., 2021; Zhang et al., 2022). One such medicinal crop is soybean.

Soybean [*Glycine max* (L.) Merr.] is a multifaceted nutritional and golden legume crop containing proteins, minerals, and other constituents (Rajendran & Lal, 2020; Ramlal et al., 2022a; Kumar et al., 2022; Mandal et al., 2022; Rajendran et al., 2022). Soybean has been widely associated with reducing BP and obesity. It shows an anti-cholesterol activity by lowering both genic and non-genic origin-based hypercholesterolemic and triglycerides, thus reducing the risk of CVD and simultaneously reducing postmenopausal symptoms and the risk of osteoporosis and antimutagenic effects (Chen et al., 2012; Handa et al., 2020). Soy proteins help in the regulation of hypercholesterolemia and improve lipid metabolism (Caponio et al., 2020). It also possesses hypotensive activities like the inhibition of ACE I and anti-microbial and anti-thrombotic activities (Arnoldi, 2013). Soybean acts as an ideal source for the identification of bioactive peptides against hypertension with other effects (Li et al., 2022). Soy proteins such as glycinin along with other products, namely, tofu, soy protein isolates, soy hydrolysate, and fermented products of soybean (douche and tofu), all have shown the presence of an inhibitory activity against the ACE (Handa et al., 2020; Xu et al., 2021; Ramlal et al., 2022c). Recently, soybean isoflavonoids, especially genistein, were shown to be used against ACE (Ramlal et al., 2022b). Inhibitory peptides involved in the inhibition of ACE are summarized by Ramlal et al. (2022c).

To explore and identify the potential inhibitory compounds, screening methods are most useful, such as molecular docking. Here, in this article, with the use of molecular docking and dynamic simulations, soybean compounds that could potentially be involved in the inhibition of ACE are being reported and that eventually would be helpful in the identification and development of novel functional food additives and useful in the design of safer drugs for ACE inhibition.

## 2 Materials and methods

### 2.1 Structure retrieval

Protein Data Bank (PDB) coordinates of the C domain (cACE; PDB ID: 1O8A) and the N domain (nACE; PDB ID: 4BZS) of ACE were retrieved from the PDB (https://www.rcsb.org/). Soybean compounds (Supplementary Figure S1) were retrieved from the PubChem (https://pubchem.ncbi.nlm.nih.gov/) database, namely, beta-sitosterol (BS; ID: 222284), soyasaponin I (SSI; ID: 122097), soyasaponin II (SSII; ID: 443614), soyasaponin II methyl ester (SSIIME; ID: 101638318), dehydrosoyasaponin I (DHSSI; ID: 656760), and phytic acid (PA; ID: 890) and other compounds (Supplementary Table S1).

### 2.2 Protein and ligand preparation

For docking experiments, the preparation of ligands was carried out using the Chimera (V: 1.15; https://www.cgl.ucsf.edu/chimera/download.html) and AutoDock Vina (V: 1.5.7) (http://vina.scripps.edu/download.html) was used for the preparation of a receptor (protein). Protein preparation was performed by using the AutoDock suite. The energy minimizations for both proteins and ligands were carried out (http://www.yasara.org/minimizationserver.htm) (Krieger et al., 2009). Heteroatoms including water were deleted, polar hydrogens were added, non-polar hydrogens were merged, and both Kollman and Gasteiger charges were added. Ligands downloaded from PubChem in an SDF format were converted into the PDB format using Open Babel (http://openbabel.org/wiki/Main_Page). Charges of the ligands were set to neutral, and Gasteiger charge was added. The number of torsions was kept as default.

### 2.3 Protein–ligand docking

Molecular docking was performed using the AutoDock Vina tool (Trott & Olson, 2010). Blind docking was performed initially followed by precision docking. The spacing angstrom was set to 1, and then, the grid box was adjusted manually to cover all the active-site residues of the receptor. The dimensions of the grid box were set as X = 30, Y = 30, and Z = 30, and the center grid box was set with the coordinates as center x = 49.257, center y = 37.37, and center z = 43.69 for cACE and center x = 28.259, center y = 13.045, and center z = 7.095 for nACE, and the grid box dimensions were X = 30, Y = 30, and Z = 30. Exhaustiveness was set to 8 for both proteins. All the dockings were performed in three replicates (Morris et al., 2009; Cosconati et al., 2010; Forli & Olson, 2012). The molecular docking was also carried out using InstaDock (Mohammad et al., 2020) (Supplementary Appendix S1).

### 2.4 Standard dynamic simulations

The dynamic simulation studies were performed using Discovery Studio V.4.0 with the enzymes (nACE and cACE) in docked states with reference captopril and BS. Standard dynamic cascades were applied where the first minimization algorithm was set to the steepest descent with maximum steps of 2000 and RMS gradient 1.0. The second minimization algorithm was set to conjugate gradient with maximum steps of 1,000 and RMS gradient 0.1. The heating phase was adjusted to possess a simulation time of 4 ps and an interval of 2 ps. The initial temperature was set to 50 and the target temperature to 300 with a maximum velocity of 2000. On the other hand, the equilibration phase was set with a simulation time of 10 ps and an interval of 2 ps. The target temperature was adjusted similar to the heating phase at 300, with a maximum velocity of 1000. The implicit solvent model was set to generalized born with a simple switching (GBSW), and the dynamics integrator protocol used leapfrog–Verlet (Huang and Leimkuhler, 1997; Im et al., 2003).

### 2.5 Visualization of poses

PyMoL (https://www.pymol.org/pymol.html) is used for the visualization and analysis of the docked poses and protein structures and for rendering the figures, while the LigPlot+ (V: 2.2.5) and Discovery Studio Visualizer (https://discover.3ds.com/discovery-studio-visualizer-download) were used to get the two-dimensional protein–ligand interactions (Laskowski & Swindells, 2011; Schrodinger, 2021). Cartoons of protein–ligand interactions were rendered in the complexes using Chimera (Pettersen et al., 2004).

## 3 Results

### 3.1 Molecular modeling studies

**FIGURE 1:**
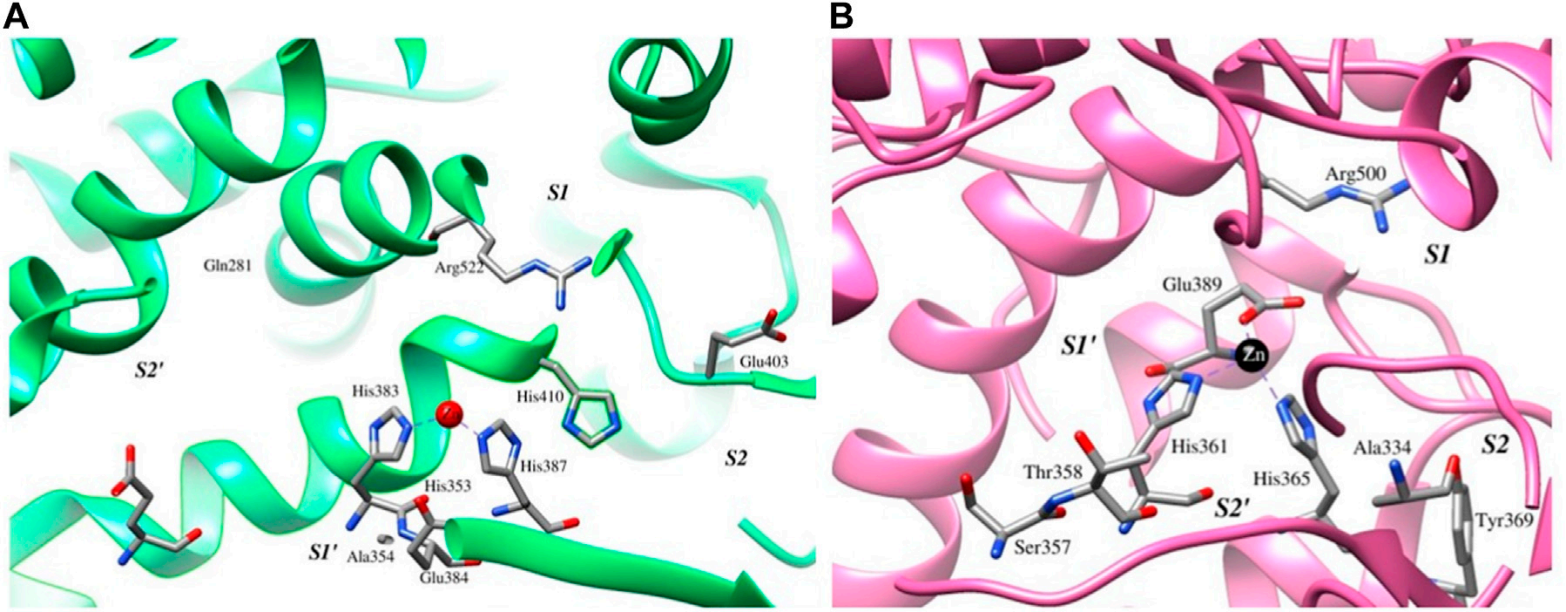
Active-site and other conserved residues are represented as sticks, enclosing the catalytic pocket and conserved HEXXH motif depicted as green and pink ribbons, and the catalytic Zn is depicted as the red and black spheres along with the subsites for **(A)** cACE and **(B)** nACE, respectively.

The binding of six compounds of soybean, namely, beta-sitosterol, soyasaponin I, soyasaponin II, soyasaponin II methyl ester, dehydrosoyasaponin I, and phytic acid (thereon BS, SSI, SSII, SSIIME, DHSSI, and PA, respectively), to the catalytic sites of both cACE and nACE was investigated using a molecular docking approach. The ACE is the ellipsoidal structure, traversed by a long and deep active-site cleft which is composed primarily of alpha-helices. The cavity is formed at the binding site which contains a ${Zn}^{2+}$ ion in its center with four subsites designated as $S_{2},S_{1},{S_{1}}^{\prime },{{and\;S}_{2}}^{\prime }$ flanking the central metal ion of the protein (receptor) while similar $P_{2},P_{1},{P_{1}}^{\prime }{{,and\;P}_{2}}^{\prime }$ designate the respective position of the inhibitor (ligand) (Figure 1). Figure 1 shows the different catalytic subsites based with respect to central zinc ion for cACE and nACE. The selectivity of inhibitors against the enzyme (ACE) could be correlated using the differences in the interaction patterns with the amino acid residues at the active site for the two domains, namely, C and N terminals. The catalytic pocket contains the ${Zn}^{2+}$ ion located centrally and surrounded by the key amino acid residues involved in the catalysis process. The cACE residues include His383, Glu384, and His387 while that of nACE includes His361, Glu362, and His365. The cACE residues, namely, Gln281 and Tyr520, interact with the ${P_{2}}^{\prime }$ terminal carboxylate. There are many hydrophobic interactions with the ${P_{1}}^{\prime }$ and ${P_{2}}^{\prime }$ groups from cACE residues Thr282, Ala354, Val379, Val380, His383, His387, and Glu411, while the nACE residues, namely, Ala332, Ser333, Glu362, and Tyr501, are found to be conserved (Cozier et al., 2018a; Cozier et al., 2018b; Caballero, 2020). Both domains have similar active-site residues but still possess differences in substrate specificities and chloride activation profiles (Cozier et al., 2018a).

### 3.2 Inhibitor-binding sites and their interactions

The distribution of interacting amino acid residues with the ligands (PA and BS) is shown in Table 1. The docking results for the PA–cACE complex reveal that it formed 11 direct hydrogen bonds with the Asn66 (3.14 Å ), Ser355 (3.29 Å ), Ala356 ( 3.66 Å , 3.09 Å , 3.06 Å , and 2.95 Å ), Tyr394 (3.33 Å ), His410 (3.34 Å ), and Arg522 (3.87 Å and 2.98 Å ) along with the catalytic zinc ion (3.77 Å ), which in turn coordinates with the catalytic residues His383 (2.02 Å ) and His387 (2.06) and Glu411 (1.97 Å ) via hydrogen bonds for the cACE. PA binding to cACE is further strengthened by many hydrophobic interactions with the catalytic subsite residues, namely, Tyr62, Tyr357, Phe391, and Glu403.

**TABLE 1:** Amino acid residues involved in ACE interactions with soy compounds forming direct hydrogen bonds and hydrophobic interactions (^a^—active-site residues).

Soy compound	**Hydrogen bond (2.0–4.0 Angstrom)**		**Hydrophobic interaction (within 4 Angstrom)**	
	nACE	cACE	nACE	cACE
**Beta-sitosterol**	His361^a^	Ala356	Val36	Glu384
			Ser39	His387
			Glu54	
			Ala332	Gln123
			Ala334	Met223
			Trp335	Ser355
			Ser353	Tyr360
			Asn494	Phe391
			Thr496	Tyr394
			Tyr501	Glu403
				Gly404
				Asn406
				Phe407
				His410
				Glu411
				Arg522
				Phe570
**Phytic acid**	Gln259	Asn66	Asp35	Tyr62
	Ser260	Ser355	Thr358	Trp357
	Glu262	Ala356	Phe435	Phe391
	His331	Tyr394	Tyr501	Glu403
	Ala332	His410	Phe505	
	Arg350	Arg522		
	Ser357			
	Glu362			
	Lys489			
	His491			
	Tyr498			

**FIGURE 2:**
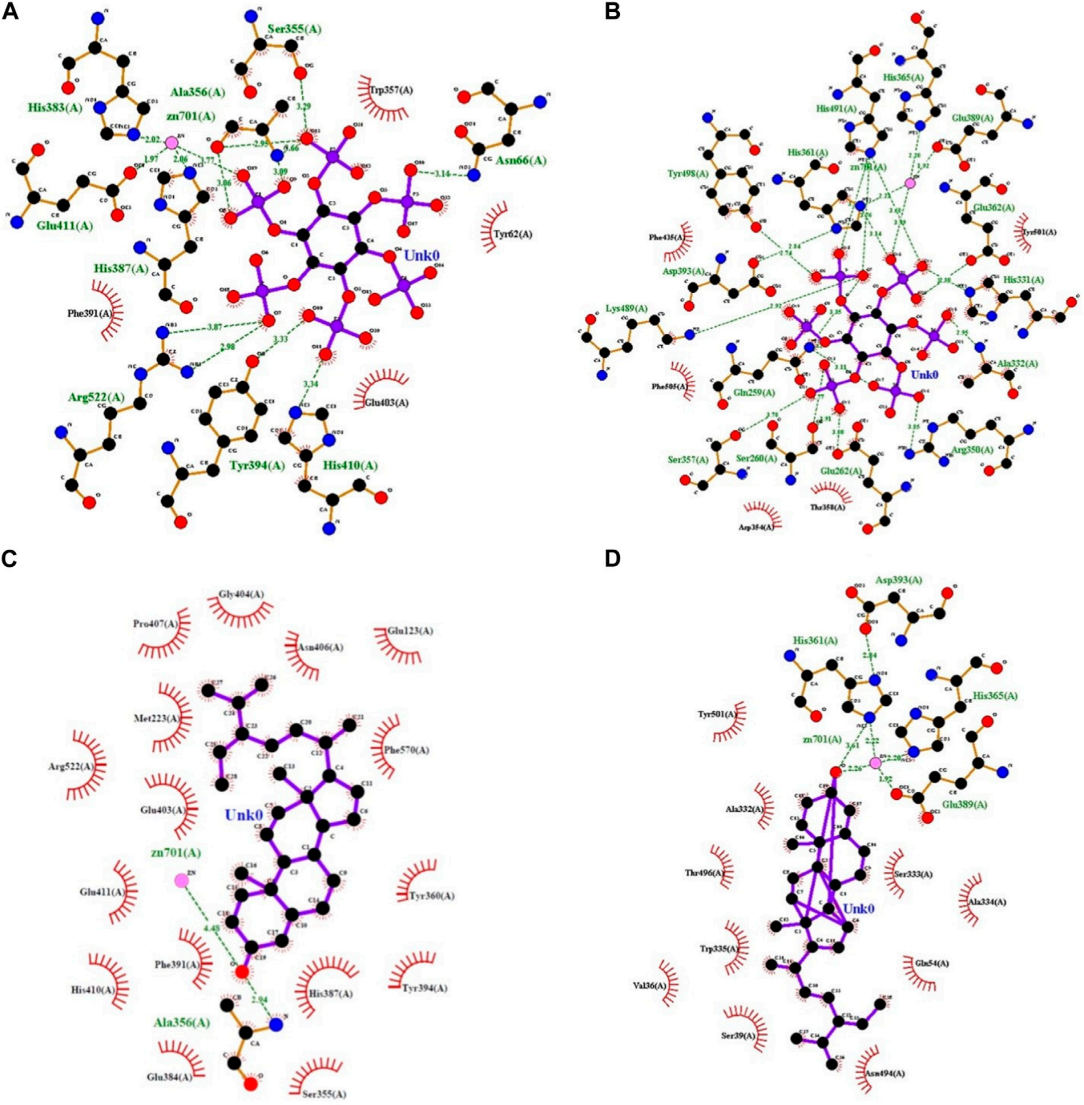
LigPlot representations of phytocompound interactions with catalytic site residues of PA-cACE **(A)**, PA-nACE **(B)**, BS-cACE **(C)**, and BS-nACE **(D)** (H-bond/electrostatic interactions are shown in green, and the residues which are involved only in hydrophobic interactions are represented by red semicircles).

For cACE, the amino acid residues His410 and Ala354 are positioned in the $S_{2}$ subsite pocket and forming hydrogen bonds with the oxygen 11 (O11) of the inhibitor, while the $S_{1}$ subsite contains residue Arg522 which is associated with oxygen 7 (O7) forming two hydrogen bonds with the inhibitor, respectively (Figure 2A).

The nACE–PA binding involves fifteen hydrogen bonds with the Gln259 (3.11 Å and 3.35 Å ), Ser260 (3.91 Å and 2.77 Å ), Glu262 (3.00 Å ), His331 (2.8 Å ), Ala332 (2.95 Å ), Arg350 (3.85 Å ), Glu362 (3.00 Å ), Lys489 (2.92 Å ), His491 (3.00 Å , 3.76 Å , and 3.68 Å ), and Tyr498 (2.74 Å ), and binding of amino acid residues with the catalytic zinc ion includes His361 (2.22 Å ), His365 (2.20 Å ), and Glu389 (1.92 Å ). There are several hydrophobic interactions with the Asp35, Thr358, Phe435, Tyr501, and Phe505 residues of nACE which is further stabilized by the binding of the ligand (Figure 2B). For nACE, the ${S_{2}}^{\prime }$ subsite has Ser357 bound with the O21. Therefore, it can be anticipated from the phytic acid-binding mode that it could act as one of the potential candidates which can work against both the C and N domains of ACE based on the binding affinities with the selective subsite residues of the residues of the catalytic pocket.

In the case of BS, the binding with cACE involves only one hydrogen bond at the site other than catalytic subsites, namely, Ala356 (2.94 Å ), while only a weak interaction (4.48 $\left.Å\right)$ with the zinc ion was observed (Figure 2C). While there were catalytic site residues coordinated with the zinc ion, 16 hydrophobic residues involved in stabilization with the bound ligand are shown in Table 1.

For the nACE–BS binding, the ligand formed only one hydrogen bond with a catalytic residue His361 with a 3.61 Å hydrogen bond with strong chelation of the zinc ion (2.26 $\left.Å\right)$ , while spanning the subsite pockets of the protein through coordinated bonds with Zn. His361, His365, and Glu389 coordinated with the catalytic zinc with bonding distances of 2.22, 2.20, and 1.92 Å , respectively, while His361 also forms a hydrogen bond with Asp393 with a 2.84 Å bonding distance. The BS binding is stabilized by various hydrophobic contacts with Val36, Ser39, Glu54, and other residues, as shown in Table 1; Figure 2D. The nACE–BS binding is stabilized by two hydrogen bonds with His361 and Glu389 at the ${S_{2}}^{\prime }$ and ${S_{1}}^{\prime }$ pockets, respectively. The predicted binding affinity of beta-sitosterol seems to be comparatively less potent to inhibit the cACE, but it can also be used for the inhibition of the nACE as well.

All 34 compounds and three reference compounds were subjected to docking analysis using InstaDock and presented a binding affinity within the range of −5.4 kcal/mol (Cap) to −15.1 kcal/mol (Urs) toward cACE and for nACE ranged from −5.3 kcal/mol (Cap) to −15.5 kcal/mol (Urs). The binding affinities and other docking parameters of each compound used in this study are shown in Supplementary Tables S2, S3. Both the dockings revealed that the binding affinities of the reference compounds, namely, quinapril, lisinopril, and captopril, with cACE and nACE were −9.5, −8.6, and −5.4 kcal/mol and −8.7, −7.5, and −5.3 kcal/mol, respectively.

### 3.3 Standard dynamic simulations

**FIGURE 3:**
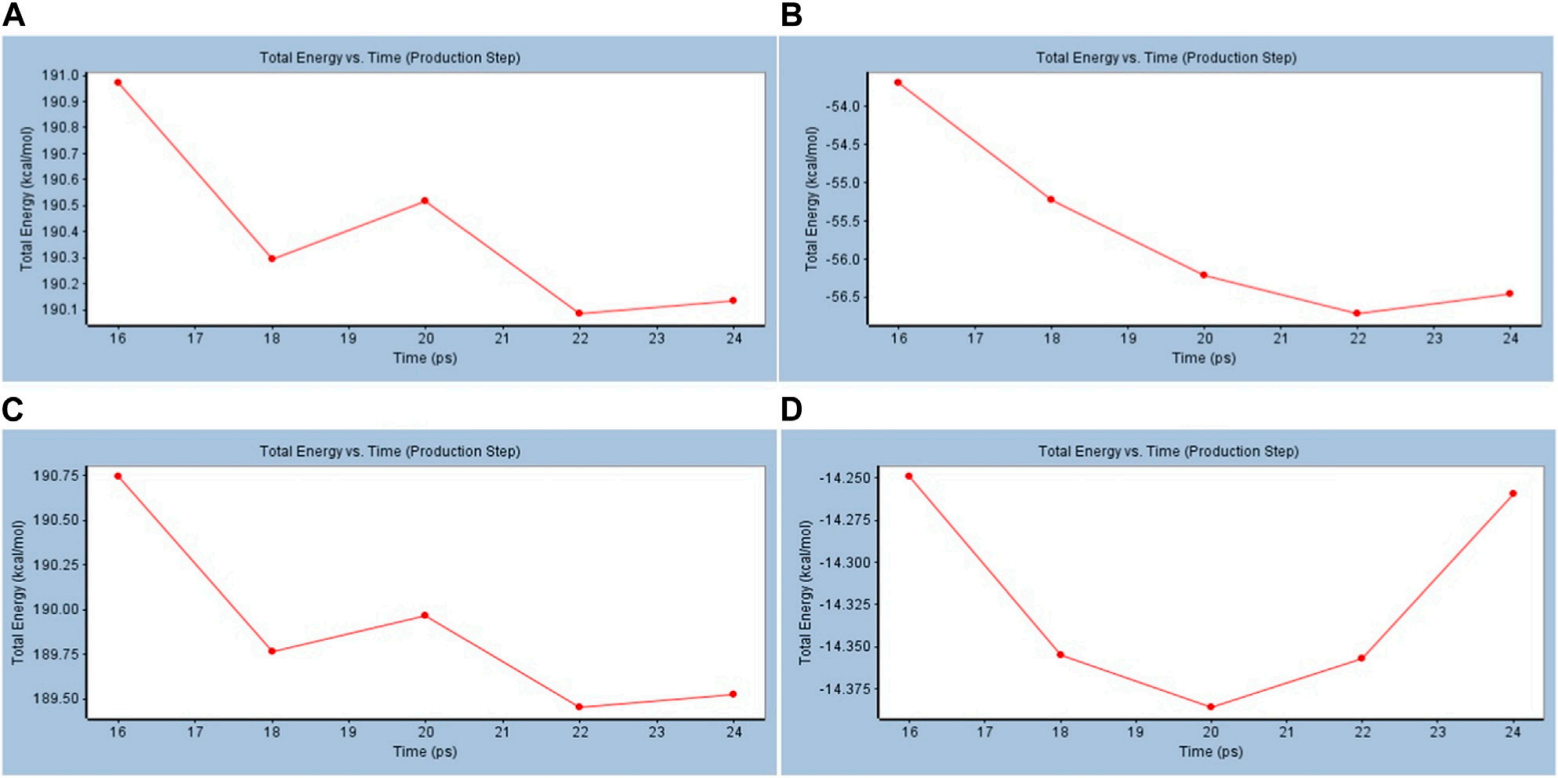
Standard dynamic simulation depicting the total energy versus time in the production step using beta-sitosterol. **(A)** BS vs. cACE, **(B)** captopril vs. cACE, **(C)** BS vs. nACE, and **(D)** captopril vs. nACE.

To investigate further, standard dynamic simulations (DSs) and analyzed trajectory studies were performed using the Discovery Studio 4.0 to confirm the nature of the stability of both cACE and nACE and the produced conformations after docking with the most active promising compound compared to reference captopril and their free states. The stability could be reflected *via* total energy calculations versus time and root mean square deviation (RMSD) versus conformations. The total energy decreases with time in the case of BS–cACE, while against the same time frame docked with the enzyme and captopril, the total energy decreases as well (Figure 3). Similarly, for nACE, the simulation studies showed a decrease in the total energy versus time with both BS and captopril and a slight increase after 22 ps in BS vs. nACE while there is a steep increase with captopril vs. nACE (Figure 3) (features of the run are shown in Supplementary Tables S4, S5).

### 3.4 RMSD analysis

**FIGURE 4:**
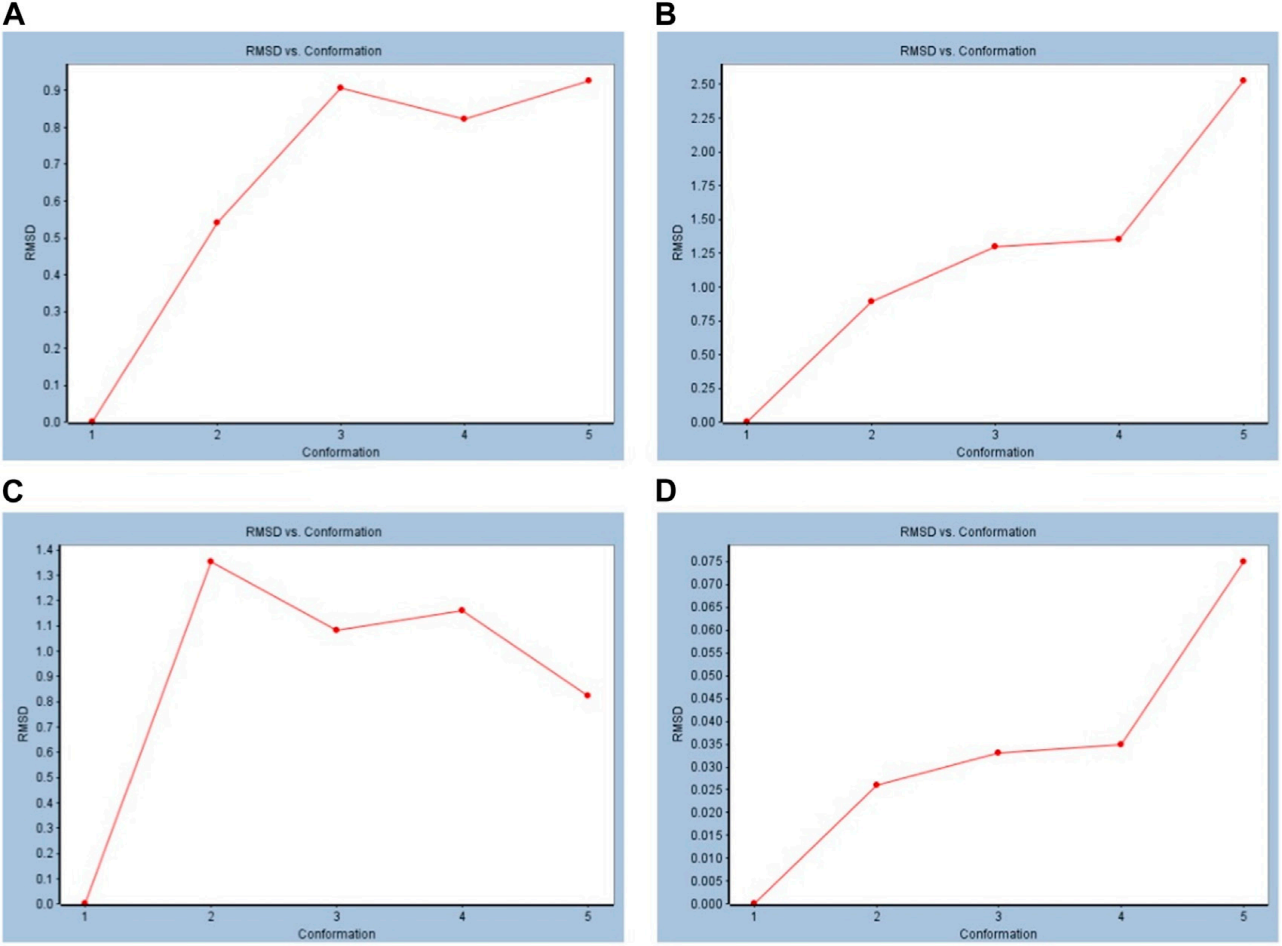
Standard dynamic simulation depicting the RMSD versus time in the production step using beta-sitosterol. **(A)** BS vs. cACE, **(B)** captopril vs. cACE, **(C)** BS vs. nACE, and **(D)** captopril vs. nACE.

The value of RMSD is an indicator of the stability of the receptor–ligand complex. The RMSD versus conformation results showed that in both BS and captopril with cACE, the RMSD increased (Figure 4). Similarly, in the simulations with the nACE, studies showed an increase in the RMSD with both BS and captopril (Figure 4).

## 4 Discussion

Despite the availability of commercial drugs for ACE inhibition, the lower success rates of drugs and prolonged treatment procedures with persistent side effects (angioedema and cough) and with no one-time remedy (Sharma et al., 2016) necessitate the search for natural solutions against it. Moreover, since the outbreak of the pandemic and even before its occurrence, herbal medicines were the preferred choice over synthetic drugs due to their side effects (Karimi et al., 2015; Farooq and Ngaini, 2021). Therefore, phytocompounds are being searched for their inhibitory activity against ACE as an alternative therapy. In the current study, different compounds of soybean were used to investigate and study the interactions in the search of the inhibitory activity for selected compounds and predicting binding affinities with both the ACE domains, namely, cACE and nACE, through the *in silico* approach. The compounds studied, namely, beta-sitosterol, soyasaponin I, soyasaponin II, soyasaponin II methyl ester, dehydrosoyasaponin I, and phytic acid, were found to be effective in controlling hypertension and cardiovascular diseases (Isanga and Zhang, 2008; Swallah et al., 2022). The compounds presented in this study are a small subset and were randomly chosen from the bioactive compounds (Supplementary Figure S1) of soybean known to be involved in controlling CVDs.

Among the docked compounds, phytic acid was found to be more potent as compared to the rest of the compounds in terms of more hydrogen bonds (10 and 17 with cACE and nACE, respectively) than BS (two each) interactions with residues at ACE catalytic subsites ensuring a stronger binding with respect to molecular docking. It is observed that phytic acid is a moderate cACE and nACE inhibitor, while the other beta-sitosterol exhibited selective inhibition profiles for the N domain of ACE. However, BS is observed to be stable with the DS analysis and formed 83 new hydrogen bonds ranging from 1.413–1.09 Å . The dockings of BS: cACE and BS: nACE showed that the binding energies are −9.2 and −9.8 kcal/mol, respectively. The molecular interactions between these compounds and the C and N domains of the enzyme were predicted and analyzed through molecular docking. The potential of the identified ligand was further confirmed by MD simulation. Therefore, it is hypothesized that soybean phytocompounds may act as good and potential inhibitors against the enzymes. To our best knowledge, this work sheds light on the potential candidates to inhibit the action of the soy compounds on binding to c- and n-ACEs.

## 5 Conclusion and future prospects

ACE is a key enzyme in the RAAS which helps in the regulation of hypertension. The overproduction of angiotensin by the activity of ACE leads to a medical condition known as hypertension, and consumption of synthetic drugs causes many side effects and sometimes even death. Therefore, it becomes very important to control and inhibit hypertension and ACE using natural compounds such as phytocompounds like saponins, terpenes, and isoflavonoids. In the current study, the molecular basis of the selectivity of some soy compounds as candidate ACE inhibitors was determined through *in silico* molecular-docking approaches. The rest of the compounds (Supplementary Table S1) need to be evaluated in detail. Overall, among the interactions studied using the *in silico* and MD approaches, the BS confirmed the stability and indicated its potential to be developed as an ACE drug to combat hypertension and cardiovascular diseases. The results obtained in this study need to be confirmed and validated through experiments and *in vivo* studies, and also, they must go through proper preclinical and clinical trials for further scientific validation. Moreover, natural compounds and other phytoconstituents should be searched for their inhibitory activity against ACE for a safer alternative and future drug design, and this study will serve as a starting point in this direction.

## Data Availability

The original contributions presented in the study are included in the article/Supplementary Material; further inquiries can be directed to the corresponding author.
