# Supplementary material for: In silico analysis of angiotensin-converting enzyme inhibitory compounds obtained from soybean [Glycine max (L.) Merr.]
Source: Front Physiol. 2023 May 31;14:1172684. doi: 10.3389/fphys.2023.1172684 (PMC10264776; doi:10.3389/fphys.2023.1172684)
Supplement: Supplementary file 1 [file DataSheet2.pdf]

# Supplementary Appendix

## Molecular docking using InstaDock

## Materials and Methods

Virtual screening of a library of 37 compounds (34 soy compounds and 3 reference compounds) with both cACE and nACE was performed to predict their binding affinity and detailed interactions. The docking was performed using InstaDock, a single-click molecular docking tool that automizes the entire process of molecular docking-based virtual screening (Mohammad et al., 2020). The binding affinities between the ligand and protein were calculated using the QuickVina-W (Hassan et al., 2017) (Modified AutoDock Vina (Trott and Olson, 2010) program which uses a hybrid scoring function (empirical + knowledge-based) in docking calculations and a blind search space for the ligand.

The p*Ki*, the negative decimal logarithm of the inhibition constant (Shityakov and Förster, 2014) was calculated from the $\Delta G$ parameter while using the following formula:

$$\Delta G = \mathrm{RT}(\mathrm{Ln}\ Ki_{\mathrm{pred}})$$

$$Ki_{\mathrm{pred}} = e^{(\Delta G/RT)}$$

$$\mathrm{p}Ki = -\log(Ki_{\mathrm{pred}})$$

where $\Delta G$ is the binding affinity (kcal $mol^{-1}$), R (gas constant) is 1.98 cal(mol $K)^{-1}$, T (room temperature) is 298.15 Kelvin, and $Ki_{\mathrm{pred}}$ is the predicted inhibitory constant.

Ligand efficiency (LE) is a commonly applied parameter for selecting favorable ligands by comparing the values of average binding energy per atom (Hopkins et al., 2004). The following formula was applied to calculate LE:

$$\mathrm{LE} = -\Delta G/\mathrm{N}$$

where LE is the ligand efficiency (kcal $mol^{-1}$ non-H $atom^{-1}$), $\Delta G$ is binding affinity (kcal $mol^{-1}$) and N is the number of non-hydrogen atoms in the ligand.
