# Supplementary material for: In silico analysis of angiotensin-converting enzyme inhibitory compounds obtained from soybean [Glycine max (L.) Merr.]
Source: Front Physiol. 2023 May 31;14:1172684. doi: 10.3389/fphys.2023.1172684 (PMC10264776; doi:10.3389/fphys.2023.1172684)

Supplementary Tables

S No	Soybean compounds (Abbreviation)	CID	Ref
1	Epoxidized Soybean oil (ESO)	71306824	PubChem
2	Soybean Lecithin (Lec)	57369748	PubChem
3	6"-O-Acetyldaidzin (AD)	156155	Isanga and Zhang, 2008; Swallah et al., 2022
4	6"-O-Acetylgenistin (AGen)	5315831	Isanga and Zhang, 2008; Swallah et al., 2022
5	6"-O-Acetylglycitin (AGly)	10228095	Isanga and Zhang, 2008; Swallah et al., 2022
6	6"-O-Malnoyldaidzin (MD)	9913968	Isanga and Zhang, 2008; Swallah et al., 2022
7	6"-O-Malnoylgenistin (MGen)	15934091	Isanga and Zhang, 2008; Swallah et al., 2022
8	6"-O-Malnoylglycitin (MGly)	23724657	Isanga and Zhang, 2008; Swallah et al., 2022
9	Chlorogenic acid (CA)	794427	Lim et al., 2021
10	Cinnamic acid (CiA)	444539	Lim et al., 2021
11	Ferulic acid (FA)	445858	Lim et al., 2021
12	Gentisic acid (GA)	3469	Lim et al., 2021
13	Syringic acid (SyA)	10742	Lim et al., 2021
14	Vanillic acid (VA)	8468	Lim et al., 2021
15	Lignan (Lig)	261166	Lim et al., 2021
16	Ursane (Urs)	9548870	Lim et al., 2021
17	Dammarane (Dam)	9548714	Lim et al., 2021
18	Cycloartane (Cyc)	160497	Lim et al., 2021
19	soyasapogenol A (SSA)	12442849	Lim et al., 2021
20	soyasapogenol B (SSB)	115012	Lim et al., 2021
21	soyasapogenol E (SSE)	13632872	Lim et al., 2021
22	Linoleic acid (LA)	5280450	Lim et al., 2021, PubChem
23	9-cis, 12-cis-octadecadienoic acid (10 – Nitrolinoleic acid) (NLA)	5282259	Lim et al., 2021
24	9-cis, 11-trans-octadecadienoic acid (LA1)	91712818	Lim et al., 2021
25	Pinitol (D-Pinitol) (Pin)	164619	Lim et al., 2021
26	Beta-sitosterol (BS)	222284	Isanga and Zhang, 2008; Swallah et al., 2022
27	Soyasaponin I (SSI)	122097	Isanga and Zhang, 2008; Swallah et al., 2022
28	Soyasaponin II (SSII)	443614	Isanga and Zhang, 2008; Swallah et al., 2022
29	Soyasaponin II methyl ester (SSIIME)	101638318	Isanga and Zhang, 2008; Swallah et al., 2022
30	Dehydrosoyasaponin I (DHSSI)	656760	Isanga and Zhang, 2008; Swallah et al., 2022
31	Phytic acid (PA)	890	Isanga and Zhang, 2008; Swallah et al., 2022
32	Genistein (Gen)	5280961	Ramlal et al., 2022b
33	Daidzein (Dai)	5281708	Ramlal et al., 2022b
34	Glycitein (Gly)	5317750	Ramlal et al., 2022b

35	Quinapril (Qin)	54892	Reference
36	Lisinopril (Lin)	5362119	Reference
37	Captopril (Cap)	44093	Reference

Table S1: Compounds used in the current study.

Name of the ligand	Binding Free Energy (kcal/mol)	pKi	Ligand Efficiency (kcal/mol/non-H atom)	Torsional Energy
Urs	-15.1	11.08	0.5033	0
SSE	-14	10.27	0.4242	0.9339
SSB	-13.7	10.05	0.4152	1.2452
SSA	-13.5	9.9	0.3971	1.5565
BS	-10.1	7.41	0.3367	1.2452
MD	-10.1	7.41	0.2525	3.7356
Dam	-9.8	7.19	0.3267	1.5565
MGly	-9.8	7.19	0.2333	4.0469
AD	-9.7	7.11	0.2622	3.113
AGen	-9.7	7.11	0.2487	3.4243
AGly	-9.6	7.04	0.2462	3.4243
Qin	-9.5	6.97	0.2794	3.113
MGen	-9.5	6.97	0.2262	4.0469
Cyc	-8.7	6.38	0.29	1.5565
Lin	-8.6	6.31	0.2687	4.0469
Dai	-8.5	6.23	0.4048	0.9339
Gen	-8.4	6.16	0.3652	1.2452
CA	-8.1	5.94	0.27	3.113
ESO	-8.1	5.94	0.1174	15.565
Gly	-8	5.87	0.3478	1.2452
SyA	-7.6	5.57	0.5429	1.5565
Lig	-7.6	5.57	0.2303	2.8017
PA	-7.4	5.43	0.1762	5.6034
SSII	-7.1	5.21	0.0973	5.2921
DHSSI	-7.1	5.21	0.0947	5.6034
VA	-6.9	5.06	0.575	1.2452
Lec	-6.9	5.06	0.1568	9.9616
LA1	-6.8	4.99	0.2833	4.3582
SSIIME	-6.3	4.62	0.0851	5.6034
FA	-6.1	4.47	0.4067	1.2452
NLA	-6.1	4.47	0.2652	4.6695
LA	-5.9	4.33	0.295	4.3582
CiA	-5.8	4.25	0.5273	0.6226
SSI	-5.7	4.18	0.075	5.9147
GA	-5.6	4.11	0.4667	0.9339
Pin	-5.6	4.11	0.3111	1.8678
Cap	-5.4	3.96	0.36	1.2452

Table S2: Binding affinities, the negative decimal logarithm of inhibition constant (pKi), ligand efficiencies and torsional energies of compounds docked with cACE using InstaDock (Red colored compounds show reference compounds).

Name of the ligand	Binding Free Energy (kcal/mol)	pKi	Ligand Efficiency (kcal/mol/non-H atom)	Torsional Energy
Urs	-15.5	11.37	0.5167	0
SSE	-14.8	10.86	0.4485	0.9339
SSB	-14.5	10.64	0.4394	1.2452
SSA	-14.4	10.56	0.4235	1.5565
SSII	-11.3	8.29	0.1548	5.2921
SSIIME	-10.8	7.92	0.1459	5.6034
SS1	-10.7	7.85	0.1408	5.9147
DHS1	-10	7.33	0.1333	5.6034
AGly	-9.7	7.11	0.2487	3.4243
AD	-9.6	7.04	0.2595	3.113
AGen	-9.5	6.97	0.2436	3.4243
MD	-9.5	6.97	0.2375	3.7356
MGen	-9.5	6.97	0.2262	4.0469
MGly	-9.3	6.82	0.2214	4.0469
Dam	-9.2	6.75	0.3067	1.5565
Cyc	-9	6.6	0.3	1.5565
BS	-8.9	6.53	0.2967	1.2452
Qin	-8.7	6.38	0.2559	3.113
CA	-8.5	6.23	0.2833	3.113
Gen	-8.4	6.16	0.3652	1.2452
Dai	-8.3	6.09	0.3952	0.9339
Gly	-8	5.87	0.3478	1.2452
Lin	-7.5	5.5	0.2344	4.0469
PA	-7.4	5.43	0.1762	5.6034
SyA	-7.2	5.28	0.5143	1.5565
Lig	-7.1	5.21	0.2152	2.8017
Va	-6.8	4.99	0.5667	1.2452
ESO	-6.8	4.99	0.0986	15.565
Lec	-6.4	4.69	0.1455	9.9616
LA1	-6.3	4.62	0.2625	4.3582
NLA	-6.2	4.55	0.2696	4.6695
FA	-6.1	4.47	0.4067	1.2452
Pin	-5.8	4.25	0.3222	1.8678
LA	-5.7	4.18	0.285	4.3582
CiA	-5.6	4.11	0.5091	0.6226
GA	-5.6	4.11	0.4667	0.9339
Cap	-5.3	3.89	0.3533	1.2452

Table S3: Binding affinities, the negative decimal logarithm of inhibition constant (pKi), ligand efficiencies and torsional energies of compounds docked with nACE using InstaDock (Red colored compounds show reference compounds).

Stage	Start Time (ps)	End Time (ps)	Initial Potential Energy (kcal/mol)	Total Energy (kcal/mol)	Potential Energy (kcal/mol)	Kinetic Energy (kcal/mol)	Temperature (K)	Van der Waals Energy (kcal/mol)	Electrostatic Energy (kcal/mol)	Initial RMS Gradient (kcal/(mol x A))	Final RMS Gradient (kcal/(mol x A))
**Minimization**			79.71		79.224			-15.414	8.891	0.802	0.462
**Minimization2**			79.224		78.346			-15.93	8.938	0.462	0.096
**Heating**	0	4	78.346	98.233	88.234	9.998	54.687	-14.498	8.959	1.736	7.283
**Equilibration**	4	14	88.234	190.578	137.423	53.155	290.75	-11.464	9.019	7.283	17.123
**Production**	14	24	137.423	189.524	132.899	56.625	309.726	-9.283	9.183	17.123	16.692

Table S4: Characteristic features of the MD of the cACE-BS. CHARMm force field was employed for the cACE (protein) and BS (ligand).

Time PS	Step	Kinetic energy	Potential energy	ep-k	Temperature	Bond energy	Angle energy	Torsion energy	Improper torsion energy	van der waals energy	Electrostatic energy	Hydrogen bond energy	RoG
**16**	8,000	48.8702	141.872	93.0018	267.311	12.7853	59.1803	75.8883	0.6347	-7.9235	8.7971	0	0
**18**	9,000	61.2525	128.513	67.2601	335.04	11.3308	52.2983	70.8639	4.1294	-11.5799	8.8331	0	0
**20**	10,000	56.1221	133.841	77.7186	306.978	15.702	52.9127	74.3954	1.4934	-12.1777	9.0309	0	0
**22**	11,000	57.6911	131.762	74.0709	315.559	11.8129	55.4058	73.0151	0.524	-9.9534	8.6435	0	0
**24**	12,000	56.6247	132.899	76.2746	309.726	7.8694	57.8915	70.1436	4.3231	-9.2826	9.1834	0	0

Table S5: MD of the cACE-BS showing energy, temperature, radius of gyration restrain energy (RoG) including other parameters of the run.

Figure(s)

Figure S1: Bioactive compounds from soybean and reference compounds used in the current study (refer to Table S1 for compound names).

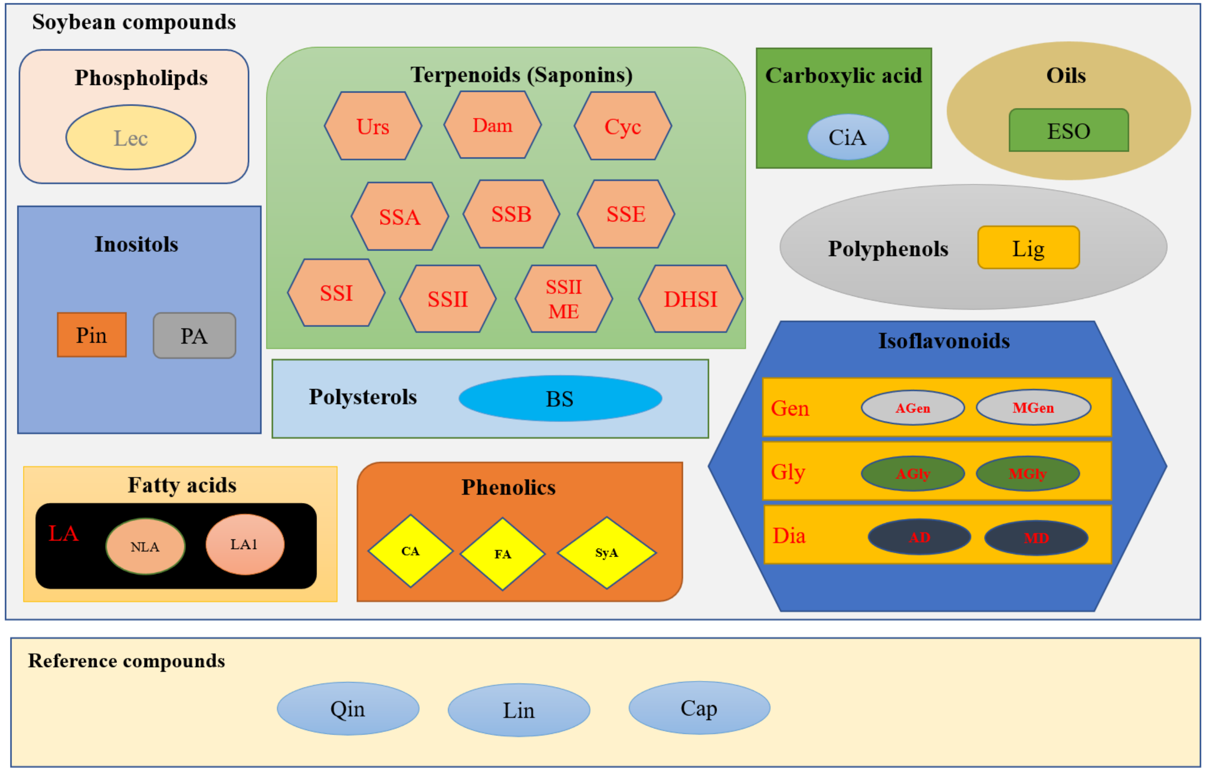

Supplement: Supplementary file 2 [file DataSheet1.pdf]
